# Impact of counterion and salt form on the properties of long-acting injectable peptide hydrogels for drug delivery†

**DOI:** 10.1039/d4fd00194j

**Published:** 2025-01-03

**Authors:** Jessica V. Moore, Emily R. Cross, Yuming An, Sreekanth Pentlavalli, Sophie M. Coulter, Han Sun, Garry Laverty

**Affiliations:** a Biofunctional Nanomaterials Group, School of Pharmacy, Queen’s University Belfast, Medical Biology Centre 97 Lisburn Road Belfast Northern Ireland BT9 7BL UK garry.laverty@qub.ac.uk

## Abstract

Modifying the salt form of active pharmaceutical ingredients is a common method to enhance their physicochemical and biological properties, whilst improving their ability to be formulated into medicines that can be effectively delivered to patients. Salts and counterions are especially relevant to peptide therapies, given that the majority of low molecular weight peptides synthesised by solid-phase protocols form a trifluoroacetate (TFA) salt due to the use of trifluoroacetic acid in resin cleaving and follow-on purification methods. TFA salts are not viewed as favourably by medicine regulators and can be defined as a new chemical entity entirely due to their different biological and physicochemical properties. Despite some exceptions, the vast majority of therapeutic peptides are marketed as hydrochloride (HCl) or acetate salts, even though most early research and development is centred on TFA salts. The aim of the study was to compare the impact of salt form (TFA *vs.* HCl) on the biostability, cell cytotoxicity, drug release and rheological properties of a Napffky(p)G-OH peptide hydrogel platform that demonstrates promise as a long-acting drug delivery system. This study demonstrated no significant difference between the salt forms for properties important to its intended use. This paper also raises important points for discussion relating to the environmental and regulatory status of peptide salts and their use as pharmaceuticals.

## Abstract 2

The study compares the impact of salt form (TFA *vs.* HCl) on the biostability, cell cytotoxicity, drug release and rheological properties of a Napffky(p)G-OH peptide hydrogel platform that demonstrates promise as a long-acting drug delivery system.

## Graphical abstract

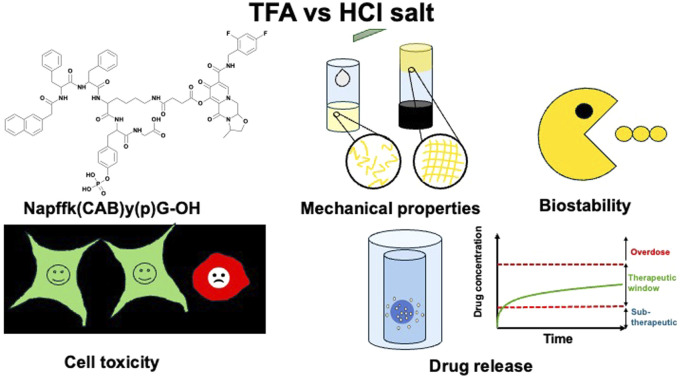

## 1. Introduction

Pharmaceutical salts are ionisable drugs or active pharmaceutical ingredients that form a neutral complex after being combined with a counterion.^1^ Approximately half of all marketed drugs exist as pharmaceutical salts due to the benefits they can provide in improving the physical and chemical properties, for example aqueous solubility, of the active pharmaceutical ingredient.^2,3^ Generic manufacturers of off-patented drugs commonly produce different salt forms of drugs once patent protection ceases as they can be cheaper and more amenable to synthesise, formulate and manufacture. Different salt forms of the same active pharmaceutical ingredient are defined as “pharmaceutical alternatives”. Medicine regulators, including the UK’s Medicines and Healthcare products Regulatory Agency (MHRA), the United States’ Food and Drug Administration (FDA) and the European Medicines Agency (EMA), require pharmaceutical alternatives to establish bioequivalence to their original product in order to obtain a marketing authorisation. This enables a marketing license to be obtained for use in the wider patient population.^4–6^ Early stage bioequivalence and pharmacokinetic studies are essential in determining the fate of any new drug product given the time, expense and high rates of failure associated with the drug development process. Bioequivalence tends to be established through a shorter clinical trial, less detailed than for the original licensed product, focusing on pharmacokinetic parameters such as maximum blood serum concentration (*C*_max_), the time taken to reach this maximum blood serum concentration (*t*_max_) and the total drug exposure over time, which is defined as the area under the curve (AUC) based on serum drug concentration as a function of time.

The existence of pharmaceutical salts is especially relevant to peptide medicines due to their increasing popularity in drug development and the formation of trifluoroacetate (CF_3_COO^−^) salts from standard methods of manufacture *i.e.*, solid-phase peptide synthesis protocols.^7^ Within currently employed methods trifluoroacetic acid is commonly used to (i) remove the final peptide sequence from its solid-phase resin support and (ii) as part of the final purification process as a constituent of the mobile phase (∼0.1% v/v) of chromatographic purification methods.

Trifluoroacetate (TFA) salts are not viewed as favourably by medicine regulators as other salts due to concerns relating to their potential toxicity. Different salts of the same peptide or drug can be defined as new chemical entities entirely due to the impact of salt form on a peptide/drug’s biological and physicochemical properties *e.g.* aqueous solubility.^8^ For such reasons the majority of peptide medicines are marketed as hydrochloride (HCl) or acetate salts.^9^ This is despite most preliminary lab-scale research being focused on TFA salts in order to reduce loss of product due to additional salt conversion steps (TFA → HCl or acetate) and to increase the quantity of peptide available for further study and characterisation. TFA salts of marketed peptide pharmaceuticals do exist, therefore demonstrating the use of TFA salts is not a barrier to clinical use in patients so long as their safety, quality and efficacy are proven to regulators. Examples include the prothrombin inhibitor bivalirudin (Angiomax^®^/Angiox^®^) and corticorelin (Acthrel^®^), the synthetic analogue of human peptide corticotropin-releasing factor.^9^ TFA is also a metabolite of the commonly employed anaesthetics desflurane, halothane and isoflurane.^10^ Therefore questions remain with regards to the need for converting peptides to HCl or acetate salts now that the pharmaceutical market is beginning to see more TFA peptide medicines approved. Would developing peptide medicines more widely as TFA salts support improved ease and speed of manufacture and reduce overall drug development cost?

Peptide-based supramolecular hydrogels are promising materials which have the potential to be utilised throughout medicine, including for the treatment and prevention of bacterial,^11^ viral,^12^ and fungal infections,^13^ and as 3D cell culture platforms.^14^ Given their importance to drug development, the nature of peptide counterion and its corresponding salt form will likely impact the clinical translation of peptide hydrogel platforms. Our research group is studying the use of self-assembling peptide hydrogels as long-acting injectable drug delivery platforms.^15,16^ This technology has the potential to improve patient adherence to medicines within several clinical areas, including schizophrenia and pre-exposure prophylaxis (PrEP) against HIV infection. Given the influence of counterions on the physicochemical and biological properties of peptides, the objective of this preliminary study was to establish whether different salt forms (TFA *vs.* HCl) would significantly impact properties important for the use of peptides as long-acting formulations (biostability, cell cytotoxicity, rheological properties, drug release). We utilised a phosphorylated d-peptide enantiomer capable of forming supramolecular hydrogels in response to phosphatase enzymes and delivering the HIV antiretroviral zidovudine to clinically relevant levels *in vivo* for 35 days.^15^ For this study we combined this peptide, (naphthalene-2-ly)-acetyl-d-phenylalanine-d-phenylalanine-d-lysine-phosphorylated d-tyrosine-glycine-OH (Napffky(p)G-OH), with the more potent HIV integrase inhibitor cabotegravir. This drug was recently approved for HIV treatment and PrEP as the long-acting injectable nanosuspension products Vocabria and Apretude.^17^

## 2. Methods

### 2.1. Materials

Wang resin preloaded with glycine (Fmoc-Gly-Wang resin, 100/200 mesh particle size, 0.4–0.8 mmol g^−1^ loading) and Fmoc protected d-amino acids including Fmoc-*O*-phospho-d-tyrosine (Fmoc-(d)Tyr(PO_3_H_2_)-OH), Fmoc-(d)Phe-OH, Fmoc-(d)Lys(Boc)-OH and Solutol® HS 15 were purchased from Sigma-Aldrich (Gillingham, Dorset, UK). Anhydrous dimethylformamide (DMF), 1-hydroxybenzotriazole hydrate (HOBt), 2-(1*H*-benzotriazol-1-yl)-1,1,3,3-tetramethyluronium hexafluorophosphate (HBTU), diisopropyl ethylamine (DIPEA), methanol, anhydrous pyridine, *tert*-butylammonium fluoride (TBAF), hexane, triethylamine (TEA), imidazole, dichlorobenzoylchloride, *N*′*N*′-diisopropylcarbodiimide (DIC), piperidine, potassium cyanide, ninhydrin, phenol, 4-dimethyl-aminopyridine (DMAP), trifluoroacetic acid (TFA), dichloromethane (DCM), diethyl ether, acetone, sodium bicarbonate, anhydrous sodium sulphate, chloroform, hydrochloric acid, ethyl acetate, acetonitrile (ACN) and *N*-hydroxysuccinimide (NHS) were obtained from Fluorochem Ltd (Hadfield, UK). Succinic anhydride, benzylamine, triisopropylsilane (TIPS) and thioanisole were purchased from Alfa Aesar (Heysham, Lancashire, UK). 2-Naphthaleneacetic acid (Nap) was obtained from Tokyo Chemical Industry UK Ltd (Oxford, UK). Cabotegravir (free acid form) was donated by ViiV Healthcare (London, UK). Glassware for synthesis including sintered glass funnels and round bottomed flasks were purchased from VWR International (Leicestershire, UK).

### 2.2. Synthesis, salt conversion and identification

**Fig. 1:**
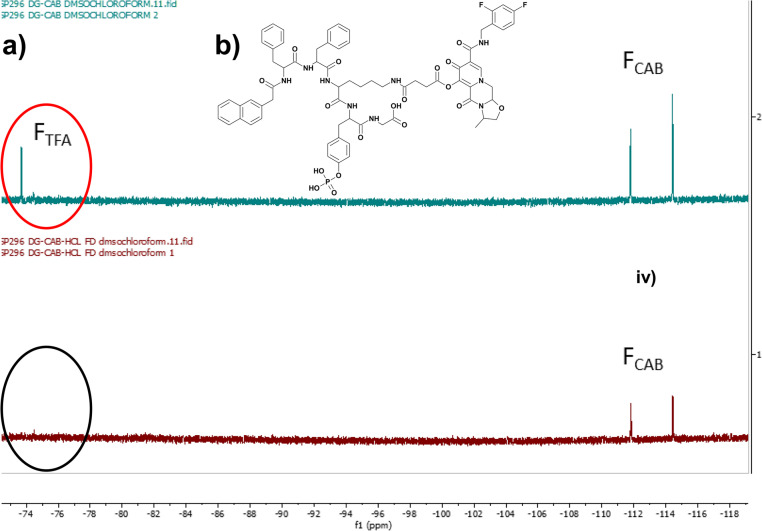
(a) ^19^F NMR demonstrates that removal of TFA was achieved by three repeated cycles of dissolving peptide in 5 mM HCl and freeze-drying. Monitoring shows removal of the singlet fluorine peak associated with TFA at −75 ppm (circled red, black circle shows removal). Key: F_TFA_^19^F NMR peaks associated with fluorine in TFA (red circle); F_CAB_^19^F NMR peaks associated with fluorine in cabotegravir. (b) Chemical structure of Napffk(CAB)y(p)G-OH.

A phosphorylated d-peptide Napffky(p)G-OH was synthesised using solid phase peptide synthetic methods as previously outlined.^15,18,19^ Wang resin preloaded with glycine (Fmoc-Gly-Wang resin) was used to ensure the peptides possessed a carboxylic acid terminus. Fmoc-*O*-phospho-d-tyrosine was incorporated onto Wang-Gly resin using a dichlorobenzoyl chloride synthetic method developed by Sieber.^20^ The HIV antiretroviral cabotegravir (CAB) was covalently attached to Napffky(p)G-OH at the ε-amino group of d-lysine (k) *via* an ester linkage, generated by the addition and subsequent activation of succinic anhydride to cabotegravir. This created a functional group that enabled precise attachment of the hydroxyl group of cabotegravir to the ε-amino side group of Napffky(p)G-OH *via* an ester cleavable linker.^15,16,21^ The chemical structure of Napffk(CAB)y(p)G-OH is shown in Fig. 1b. The purity of each product was analysed by HPLC (≥95%). The mobile phase was composed of Milli-Q ultrapure water combined with 0.1% v/v TFA and ACN. HPLC chromatograms are shown in Fig. S1 and S2.† Peptide products were lyophilised by freeze-drying using an Edwards freeze drier with an RV8 pump (Davidson and Hardy, Belfast, UK) and identified using Electrospray Time of Flight Mass Spectrometry (ESI-MS) (Waters LCT Premier, Waters, Hertfordshire, UK) performed at Analytical Services and Environmental Projects (ASEP) in the School of Chemistry, Queen’s University Belfast (Fig. S3†) and ^1^H-NMR (Bruker Ultrashield Plus 400 MHz, Bruker, Coventry, UK) (Fig. S4 and S5†). ^31^P-NMR was used to monitor the presence of the phosphate grouping within the peptide sequence (Fig. S6 and S7†). Conversion to HCl salt was achieved by three repeated cycles of dissolving the peptide in 5 mM HCl and freeze-drying as outlined previously by Andrushchenko *et al.*^22^ Removal of TFA was confirmed by ^19^F NMR by monitoring the characteristic fluorine singlet at −75 ppm (Fig. 1a).^23^

### 2.3. Biostability

The *in vitro* biostabilities of solubilised (0.02% w/v) Napffk(CAB)y(p)G-OH in TFA and HCl salt forms were tested against a broad-spectrum protease, proteinase K, at several time points for up to 28 days as previously outlined.^15,16^ Proteinase K was deactivated at each time point by removing a sample and adding concentrated acetic acid. Samples were subsequently analysed by RP-HPLC. The amount remaining (% peptide remaining) at each time point was calculated following RP-HPLC analysis as outlined in eqn (1) below.1
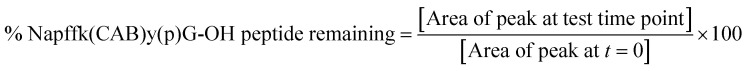


### 2.4. Mechanical properties

The propensity of TFA and HCl salts of Napffk(CAB)y(p)G-OH to gel in response to a 2 μL volume of 2 U (1000 U mL^−1^) alkaline phosphatase enzyme derived from bovine intestinal mucosa (Merck KGaA, Darmstadt, Germany) was initially screened at several concentrations (0.5–2% w/v) in pH 7.4 PBS using a vial inversion assay. Samples were incubated for 24 hours at 37 °C and propensity to gelate was defined by the formulation remaining suspended after the glass vial is inverted. Hydrogel formulation followed the steps outlined in Table S1.† The overall concentration of alkaline phosphatase enzyme within the final formulation using this method was 3.98 U mL^−1^ (2 U in 502 μL). Hydrogel formation was formally defined using oscillatory rheology performed using an Anton Paar MCR302 rheometer (Anton Paar, St Albans, UK) at 37 °C. A vane and cup geometry was used to conduct frequency (1–100 rad s^−1^, strain = 0.5%) and strain sweeps (0.1–1000%, frequency = 2.5 rad s^−1^) with TFA and HCl salts of Napffk(CAB)yG-OH formulated at a volume of 2 mL within 7 mL Sterilin™ vials (Thermo Fisher Scientific, Waltham, MA, USA). Time sweeps were performed with a constant frequency of 2.5 rad s^−1^ and a constant strain of 0.5% using a sand blasted parallel plate (PP50/S, gap size = 0.5 mm). Three replicates were used for each study.

### 2.5. Cell cytotoxicity

The cell cytotoxicities of Napffk(CAB)y(p)G-OH TFA and HCl salts were established using two methods: (i) a (3-(4,5-dimethylthiazol-2-yl)-5-(3-carboxymethoxyphenyl)-2-(4-sulphophenyl)-2*H*-tetrazolium) (MTS) CellTiter 96 AQueous One Solution Cell Proliferation Assay (Promega, Southampton, UK) to measure % cell metabolic activity compared to a negative (treated with media only) control (eqn (2)), and (ii) a Live/Dead® Viability/Cytotoxicity Fluorescence Assay (Thermo Fisher Scientific, Waltham, MA, USA) to quantify the viability of cells *via* fluorescence microscopy (EVOS FL microscope, Thermo Fisher Scientific, Waltham, MA, USA). The murine fibroblast subcutaneous tissue NCTC 929 (ATCC CCL 1) cell line was chosen for this study, as it is the accepted cell line of choice for the International Standard Organisation’s (ISO) *in vitro* biomaterial and medical device cytotoxicity testing.^24^ NCTC 929 cells were cultured to at least third passage and seeded in sterile Nunc® 96-well microtitre plates (Fisher Scientific, Leicestershire, UK) at a concentration of 1 × 10^4^ cells per well (6 hours and 24 hours treatments) and 5 × 10^3^ cells per well (72 hours treatments times). After cells were incubated for 24 hours, media was removed and 100 μL of Napffk(CAB)y(p)G-OH solution was added to separate wells across a range of concentrations (20–500 μM) for 6–72 hours timepoints as previously outlined.^15,16^2
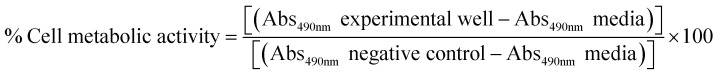


### 2.6. *In vitro* drug release

Cabotegravir release from 2% w/v Napffk(CAB)yG-OH hydrogel salts (TFA and HCl forms) were compared across 28 days under sink conditions using a release media containing 1 mL of 2% w/v Solutol® HS 15 in phosphate buffered saline(PBS), pH 7.4, at 37 °C. Drug release studies (*n* = 4) were performed in 10.5 mL glass vials and each Napffk(CAB)yG-OH hydrogel (preformed 24 hours prior) was formulated to a volume of 100 μL following the steps outlined in Table S1.† Release media (1 mL) was added on top of each gel and fractional volume sampling (600 μL supernatant replaced with 600 μL fresh release media) was performed to maintain sink conditions at each timepoint. The concentration of cabotegravir released was determined using an Agilent 1260 Series analytical HPLC system (Agilent Technologies Ltd, Cork, Ireland) fitted with a Gemini C_18_ column (250 × 4.6 mm, 5 μm particle size, 110 Å; Phenomenex, Macclesfield, UK).^25^ A calibration curve (Fig. S12†) for cabotegravir (*r*^2^ = 1) was developed as outlined within Section S.4 (ESI).† Drug release kinetics were deciphered using KinetDS 3.0 software (SourceForge Media, La Jolla, CA, USA).^26^

### 2.7. Statistical analysis

Statistical analysis was performed using GraphPad Prism 10 and Microsoft Excel 2021. The statistical difference in biostability between Napffk(CAB)y(p)G-OH·TFA and Napffk(CAB)y(p)G-OH·HCl was compared across 28 days using a two-tailed Mann–Whitney *U* test. Frequency sweeps and the impact of salt form on the storage modulus (*G*′) for Napffk(CAB)yG-OH·TFA and Napffk(CAB)yG-OH·HCl were also compared using a two-tailed Mann–Whitney *U* test. *F* tests demonstrated significant differences in variances between groups, for both biostability and frequency sweep comparisons, meaning that a non-parametric test (Mann–Whitney *U*) was employed. Statistical differences in TFA and HCl salts for the MTS cytotoxicity assay were compared at each concentration and timepoint using an unpaired *t*-test. Similarly an unpaired *t*-test was employed to compare differences with *in vitro* drug release between each peptide salt as variances between groups were similar allowing a parametric *t*-test to be utilised.

## 3. Results and discussion

### 3.1. Synthesis, salt conversion, identification and factors impacting future manufacturing upscale

Low molecular weight peptides, including Napffky(p)G-OH, are readily synthesised within the laboratory using standard Fmoc solid phase peptide synthesis protocols. A manual nitrogen bubbler system was utilised to synthesise the Napffky(p)G-OH·TFA to 0.4–0.8 mmol scale, resulting in 400–500 mg of crude peptide. The addition of succinic anhydride to cabotegravir occurs with a yield of ∼80% and enables covalent attachment of the hydroxyl group of cabotegravir to the ε-amino (d-lysine) side group of Napffky(p)G-OH. Drug attachment followed by purification with reverse phase HPLC commonly proceeds with ∼30% yield. Therefore at a laboratory scale, the synthetic steps outlined can typically be expected to produce 120–150 mg Napffk(CAB)y(p)G-OH·TFA peptide of ≥95% purity from the original 400–500 mg crude TFA containing product. Conversion to a HCl salt by three repeated cycles of dissolving peptide in 5 mM HCl and freeze-drying results in ∼65% conversion (78–98 mg) to Napffk(CAB)y(p)G-OH·HCl. Therefore, introducing an additional salt conversion step to the manufacturing process is an important consideration due to its impact on overall yield, even at laboratory-scale where synthesis of mg to lower gram quantities is most feasible.

As Fig. 1a demonstrates by the removal of the fluorine peak corresponding to TFA (labelled F_TFA_), the conversion of Napffk(CAB)y(p)G-OH·TFA to Napffk(CAB)y(p)G-OH·HCl was successfully achieved at a laboratory scale using the methods outlined.^23^ The efficacy of the synthetic process is especially relevant to supramolecular peptide gels, whereby high purity (≥95%) is required to limit any variation related to gel formation *e.g.*, mechanical properties.^27^ There is a balance between having sufficient quantity of peptide in order to fully characterise its relevant properties by high-throughput screening, and the ability to satisfy any regulatory and commercial considerations *e.g.*, intellectual property should a different peptide salt be deemed a new molecule entirely. This is especially true for medical applications where wider use in humans, from clinical trials onwards, must also be upscaled to the requirements of current Good Manufacturing Practice (cGMP) in order to meet the strict quality requirements of medicine regulators. The impact of upscaling manufacture on synthetic factors must therefore be considered within the process development phase of any pharmaceutical project involving a peptide.^28^ Such aspects include: the overall yield; raw material availability; novel *versus* established methods of synthesis and chemical conjugation of drug; chemical orthogonality; ensuring analysis to regulatory requirements (Pharmacopoeial standards); application of green chemistry; storage and distribution; and overall cost.^29^ For the cGMP manufacture of a peptide to an initial ∼100 g scale, it is likely that purification and salt conversion would be by ultra-high-pressure liquid chromatography (UHPLC) *e.g.*, using a 15 cm reverse-phase column and counterion exchange prior to lyophilisation. Any follow-on analytical considerations would also need to be defined, for example the peptide purity would not be the same as peptide content.^30^ A lyophilised powder of peptide would usually contain the peptide alongside water and the counterion (TFA, HCl, acetate). Peptide content can be measured by CHN elemental analysis, water by Karl Fischer titration, counterions by ion chromatography and residual solvents by gas chromatography.^31^ Taking into account that the majority of lyophilised peptides are hygroscopic, water content will likely increase during storage, distribution and handling.^32^ This may impact the cost of formulation, for example the need for an optimised freeze-drying process alongside suitable packaging and container compatibility studies *e.g.*, more expensive and water resistant type-I glass vials with a type-I rubber stopper, aluminium seal and polypropylene cap.

Formulation factors such as: physicochemical properties; solubility and stability; identification of degradation pathways; determination of freeze–thaw stability and the potential for aggregation must also be considered.^33^ Within this study, we were unable to fully define important physicochemical properties such as the water solubility for the different salt forms of phosphorylated Napffky(p)G-OH. Whilst this is an important pharmaceutical parameter which supports the formulations choice for each specific active ingredient *e.g.*, solution, suspension, tablet, it is a property that remains relatively undefined within supramolecular peptide gel research. Synthesising sufficient quantity of peptide on a laboratory scale to define solubility limits is challenging. It will likely be defined within the process of manufacturing upscale and through optimising formulation factors *e.g.*, lyophilisation. Throughout the peptide gel research literature, studies tend to focus on defining other factors, including critical/minimal gelation concentrations. For our intended application as an injectable product, aqueous solubility will influence how quickly a powdered peptide–drug would dissolve in a chosen water-based vehicle *e.g.*, water for injection. The nature of the freeze-drying or spray-drying process and the need for excipients *e.g.*, lyoprotective agents such as trehalose, will also have a significant influence on overall solubility.

In practice our formulation would likely be prepared as stable freeze-dried powder, which would then be readily dissolved in sterile water for injection/buffer and administered *via* syringe due to its low viscosity. As a subcutaneously administered injection, this approach would require some form of terminal sterilisation or manufacture under aseptic conditions to ensure an effectively zero bioburden. A cheaper and more user-friendly method may be to develop a pre-filled syringe that is terminally sterilised using a process such as exposure to UV radiation.^34,35^ This would remove the need for reconstitution in water/buffer immediately prior to administration by a healthcare worker, carer or patient and would be of particular value to low and middle income countries (LMICs) where healthcare infrastructure is less well developed and HIV PrEP is most needed. Pharmaceutical stability and shelf-life (preferably ≥2 years) will be a significant factor for defining the choice of formulation and ensuring that global demand for the medicine can be adequately fulfilled within a reasonable timescale. This is especially relevant to a pre-filled syringe approach, as the formulation would likely be composed mainly of water. Its impact on the peptide and drug-linker stability would need to be closely monitored across the environmental conditions (temperature, humidity) encountered across several relevant climatic zones.^36^

A further consideration is the need to both assess and reduce the environmental impact of solvents, reagents and chemicals currently employed within standard methods of peptide synthesis, using green chemistry approaches where possible. For a peptide such as Napffk(CAB)y(p)G-OH with a molecular mass between 1000–5000 Da, a significant quantity of waste would be generated. This is estimated to be between 3000–15 000 kg waste per kg of peptide manufactured, based on general estimates for peptides developed by Ferrazzano and colleagues.^37^ Such considerations are timely given the restrictions introduced on peptide synthesis solvents *e.g.*, DMF by the European Union in December 2023.^38^ DMF-free approaches to peptide synthesis have been researched, for example the use of dimethyl sulphoxide (DMSO) and ethyl acetate combinations or *N*-butylpyrrolidinone (NBP).^39,40^ However these methods require enhanced purification steps, involving larger quantities of moderately toxic solvents such as ACN and an increase in related waste products.^37^ The use of TFA has proven more difficult to replace, both as part of the mobile phase and especially for peptide cleavage from the resin in solid phase synthesis.^41^ The environmental impact of TFA is also a concern given that TFA salts are very stable and tend of accumulate within bodies of water *i.e.*, oceans, seas, lakes, rivers.^42–44^ Therefore the purification process, salt form and waste production must also be taken into account in assessing the environmental impact of small scale synthesis and large scale manufacture.

### 3.2. Propensity for gelation and biostability

**Fig. 2:**
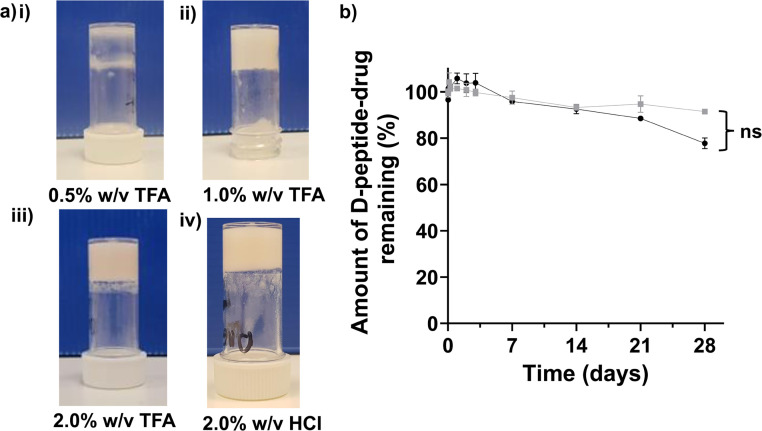
(a) Vial inversion assay performed for (i) 0.5% w/v Napffk(CAB)yG-OH (TFA salt), (ii) 1.0% w/v Napffk(CAB)yG-OH (TFA salt), (iii) 2% w/v Napffk(CAB)yG-OH (TFA salt), (iv) 2% w/v Napffk(CAB)yG-OH (HCl salt). (b) The biostability of TFA (black circles) and HCl (grey squares) salts of Napffk(CAB)y(p)G-OH after incubation with the broad-spectrum protease proteinase K for 28 days. The values represent means ± SDs (*n* = 3). Key: ns: no significant difference (*p* > 0.05) in the biostability of TFA and HCl salts over the 28 days study.

The propensity for gelation, studied by a simple vial inversion assay, demonstrated that the critical gelation concentration of Napffk(CAB)y(p)G-OH·TFA and Napffk(CAB)y(p)G-OH·HCl were ∼1.0% w/v after the addition of 3.98 U mL^−1^ of alkaline phosphatase enzyme to fully solubilised the peptides (Table S1†). This correlates to values observed in previous studies with NapFFKY(p)-OH and related peptides.^45–48^ Sufficient gelation, to allow the gel to remain suspended upon inversion of the vial, did not occur at 0.5% w/v (Fig. 2(a)i). A 2.0% w/v concentration of peptide was chosen to characterise further for mechanical and drug release properties in line with that previously employed for NapFFKY(p)-OH by the Xu group,^21^ and our own glycine containing Napffky(p)G-OH system.^15^ In practice, it is likely that peptide–drug concentrations would be increased further in order to meet the need for clinically relevant concentrations of cabotegravir to be delivered systemically to patients for ≥84 days. This can only be truly assessed when sufficient quantities of cGMP peptide have been manufactured alongside *in vivo* clinical studies *i.e.*, human trials.

Biostability is an important consideration for any peptide therapy given its links to pharmacokinetic parameters (absorption, distribution, metabolism, excretion), its ability to act at a specific target site at adequate concentrations and its corresponding efficacy. It is particularly important that sufficient biostability is obtained when intending to use a peptide as a long-acting drug delivery system for ≥84 days. As shown in Fig. 2b, the use of d-amino acids provides sufficient biostability against the model broad-spectrum protease, proteinase K, for 28 days. We previously demonstrated that the naturally occurring l-α-peptide template of NapFFKY(p)G-OH has unacceptable biostability and degrades rapidly within hours.^15^ Tests were conducted on fully solubilised peptides (0.02% w/v) in order to remove any influence viscosity and mechanical properties may have on biostability. For example viscosity is likely to impact the entry and diffusion of proteinase K throughout the peptide gel and therefore its overall ability to degrade the peptide. It is possible that the formation of a hydrogel depot clinically may be associated with improved biostability, by limiting the diffusion of protease enzymes throughout the peptide gel and lowering the accessibility of peptides to the enzyme active site. Napffk(CAB)y(p)G-OH·TFA (Fig. 2b) demonstrated a lower mean percentage of peptide remaining after 28 days incubation with proteinase K (77.8%) compared to Napffk(CAB)y(p)G-OH·HCl (91.5%). However, this difference was not found to be significant after statistical testing (Mann–Whitney *U* test) was employed. Future work will involve modifying biostability and degradation to specific drug dosage intervals *in vivo e.g.*, for 84 days. It will be important to establish this within a human context, both to ensure that the peptide has suitably degraded before administration of the next dose and that drug-release from the gel depot has been exhausted. The success of similar long-acting peptide gel platforms shows this is possible. For example the long-acting l/d peptide degarelix (Firmagon®), prescribed for treatment of metastatic prostate cancer, has a dosage interval of 28 days. Future work will focus on pharmaceutical stability (shelf-life) and the influence of salt forms on the related properties of the formulation *e.g.*, water content, physicochemical properties.^2^ Salt form has also been shown previously to significantly influence the thermal stability of peptide fibres and the formation of different supramolecular nanostructures.^49,50^ These are therefore potential areas for future research.

### 3.3. Mechanical properties

The oscillatory rheology of supramolecular gels is important to define their mechanical properties. These properties are also significant to our chosen mode of administration *i.e.*, injection, and the release of drugs from *in situ* forming hydrogels. For example, time sweeps are utilised to monitor the rheological changes encountered from a solution forming a gel over time. Quick gel formation is preferred as gel fibres will likely provide an additional diffusional barrier to minimise rapid “burst release” of drug upon administration. This is crucial to reduce the potential for side effects and toxicity associated with higher concentrations of drug, both locally and systematically, whilst ensuring that clinically relevant concentrations can be maintained throughout the required dosage interval.

A review article by the Jackson group in 2017, highlighted there were few studies relating to the influence of counterions on the kinetics or mechanisms of peptide aggregation.^51^ However, a growing body of research currently exists, mainly focused on β-amyloid plaques linked to the development of Alzheimer’s disease. The kinetics of fibril formation has been found to be more rapid in the presence of a Cl^−^ counterion, when compared to TFA. Changes in the secondary structures were also observed. For example, β-structures and fibrils form from an α-helical intermediate for TFA salts and fibrils form directly *via* β-sheet conformation for Cl^−^ salts.^9,52–54^ Such changes in secondary structures make it feasible that changing the salt form of peptides may have a significant impact on the rheological and mechanical properties of supramolecular peptide hydrogel systems given their overall effect on the kinetics of fibre formation.^55^

**Fig. 3:**
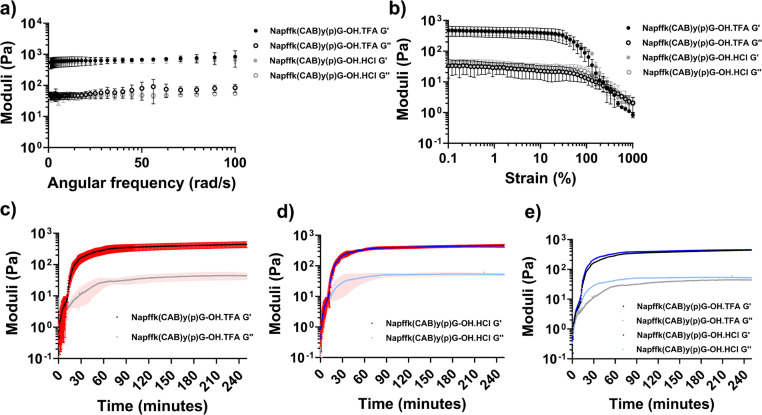
Rheological data relating to 2% w/v TFA (Napffk(CAB)y(p)G-OH·TFA) and HCl (Napffk(CAB)y(p)G-OH·HCl) salts. Means ± standard deviations (SDs) plotted for each (*n* = 3). (a) Frequency sweeps for 2% w/v Napffk(CAB)yG-OH·TFA and Napffk(CAB)yG-OH·HCl hydrogels. (b) Strain sweeps for 2% w/v Napffk(CAB)yG-OH peptide gels. In (a) and (b), Napffk(CAB)yG-OH·TFA is presented in black and Napffk(CAB)yG-OH·HCl is presented in grey. The filled circles represent the storage modulus (*G*′), and the open squares represent the loss modulus (*G*′′). (c–e) Rheological time sweeps to 250 min for (c) 2% w/v Napffk(CAB)y(p)G-OH·TFA, (d) 2% w/v Napffk(CAB)y(p)G-OH·HCl, (e) mean values for 2% w/v Napffk(CAB)y(p)G-OH·TFA and Napffk(CAB)y(p)G-OH·HCl with SDs removed to improve clarity. In (c–e) the black and dark blue lines represent the storage modulus (*G*′) for Napffk(CAB)y(p)G-OH·TFA and Napffk(CAB)y(p)G-OH·HCl respectively. The grey and light blue lines represent the loss modulus (*G*′′) for Napffk(CAB)y(p)G-OH·TFA and Napffk(CAB)y(p)G-OH·HCl respectively. The red and light red areas donate SDs for *G*′ and *G*′′ respectively.

The rheological data (Fig. 3 and Fig. S8†) demonstrated that there was no significant difference in the mechanical properties between the TFA and HCl salts of 2% w/v Napffk(CAB)y(p)G-OH. Frequency sweeps (Fig. 3a) provided an indication of gel stiffness for both Napffk(CAB)yG-OH·TFA and Napffk(CAB)yG-OH·HCl. There was no significant difference in the mean *G*′ values of 625.2 Pa (Napffk(CAB)yG-OH·TFA) and 602.2 Pa (Napffk(CAB)yG-OH·HCl) at a frequency of 1–100 rad s^−1^ (Fig. S8†). These values are within a similar range to those observed for related soft gel systems, NapFF supramolecular peptide hydrogels and that of the extracellular matrix.^11,13,15,21,45^ The attachment of cabotegravir resulted in a reduction in gel stiffness (from 4399.5 Pa for NapffkyG-OH), governed by *G*′ values obtained within frequency sweeps. Gel strength was studied *via* strain sweeps. For both TFA and HCl peptides, breakage of gels began at 36.5%. The yield point, where *G*′′ crosses *G*′ indicating total gel breakage, occurred at 333% strain.^56^

Time sweeps were performed for up to 250 minutes. As previously outlined,^15,16^ several aspects were studied to define the gelation time of the peptide salts in response to 3.98 U mL^−1^ of alkaline phosphatase enzyme. These included where *G*′ and *G*′′ initially cross, where *G*′ > 2 × *G*′′ and the time required for *G*′ to become stable (Table S2†). When taking into account each of these parameters, there was no difference in the gelation time between the peptide salts. Gels began to form within seconds of exposure to phosphatase enzyme. *G*′, dictating solid-like properties, was greater than *G*′′ (liquid character) after ∼1.67 min for both salts. *G*′ was more than double *G*′′ after 12.5 (Napffk(CAB)y(p)G-OH·TFA) and 13.8 min (Napffk(CAB)y(p)G-OH·HCl) and *G*′ began to stabilise for each after 65.3 and 62.7 min respectively. When compared to our previous studies for TFA salts of Napffky(p)G-OH alone, the covalent attachment of cabotegravir results in a slight increase in the time required for gelation, given that *G*′ and *G*′′ takes 46 minutes to stabilise for Napffky(p)G-OH·TFA.

Whilst there was no significant difference in the mechanical and rheological properties of the Napffk(CAB)y(p)G-OH·TFA and Napffk(CAB)y(p)G-OH·HCl peptides, it would be interesting to test whether differences occur at a molecular level, especially with regards to the architecture of formed fibres. For example, microscopy (AFM, TEM, SEM) and spectroscopic analysis [circular dichroism (CD), FTIR] could link mechanical strength and drug release to the fibre architecture and secondary structure of these gels. Neutron methods, for example small angle neutron scattering (SANS), ultra small angle scattering (USANS), rheo-SANS, quasi-elastic neutron scattering (QENS) and neutron imaging, could also be utilised to study how molecular packing, long-range fibre networks and water/drug diffusion are influenced by different counterions.^57–59^

### 3.4. Cell cytotoxicity

**Fig. 4:**
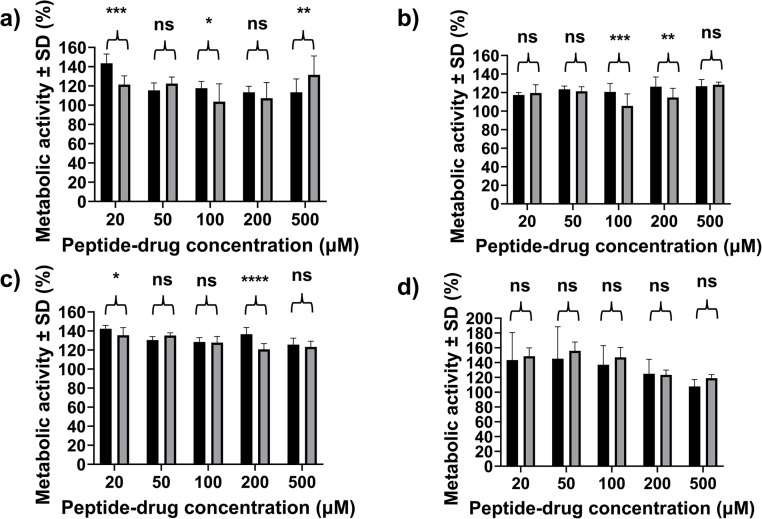
Cell cytotoxicity, represented as % metabolic activity derived from a MTS assay, after exposure to solubilised Napffk(CAB)y(p)G-OH·TFA and Napffk(CAB)y(p)G-OH·HCl at concentrations between 20–500 μM for (a) 6, (b) 24, (c) 48 and (d) 72 hours. Key: black bars: Napffk(CAB)y(p)G-OH·TFA; grey bars: Napffk(CAB)y(p)G-OH·HCl; ns: no significant difference (*p* > 0.05); *: *p* ≤ 0.05 difference; **: *p* < 0.01; ***: *p* < 0.001; ****: *p* < 0.0001 between Napffk(CAB)y(p)G-OH·TFA and Napffk(CAB)y(p)G-OH·HCl at the same concentration and timepoint.

It was important to determine any difference in toxicity between TFA and HCl Napffk(CAB)y(p)G-OH peptide salts, given the potential for different biological properties and the emphasis on safety that medicine regulators place on assessing TFA counterions in particular. In this study we tested the short-term cell cytotoxicity of TFA and HCl salts for up to 72 hours using both MTS (Fig. 4) and Live/Dead® staining (Fig. S9–S11†) assays. No significant toxicity was demonstrated for either Napffk(CAB)y(p)G-OH·TFA or Napffk(CAB)y(p)G-OH·HCl relative to the negative media only controls when concentrations of up to 500 μM were employed. When interpreting MTS data, the cell metabolic activity (%) was often higher than media only negative controls resulting in values above 100%. There was no distinct or discernible difference between cytotoxicity for TFA and HCl salts. There was no significant difference in % cell metabolic activity at the longest 72 hours timepoint at all concentrations (20–500 μM) (Fig. 4d) and there was similarity in the occurrence of green fluorescent protein, present for viable cells, visualised by Live/Dead® staining (Fig. S9–S11†). Where significant differences did occur in MTS data between salt types *e.g.*, Fig. 4b at 24 hours and Fig. 4c at 48 hours and 200 μM, these were still above 100% metabolic activity/viability and the ISO threshold acceptance criteria of 70% metabolic activity/viability for *in vitro* cytotoxicity assays.^24^ Therefore no observable toxicity was demonstrated for either peptide salt type.

Previous studies demonstrated a difference in the biological properties of TFA and HCl peptide salts. TFA salts have been previously shown to suppress the proliferation of several cell types, including chondrocytes and osteoblasts, in a dose-dependent manner.^60^ This suppression was not observed for HCl salts. Interestingly in the context of our own MTS data where an increase in cell viability often occurs, TFA salts were previously shown to induce cell growth and increase cell viability within the micromolar (μM) concentration range. This study by Ma and colleagues used murine glioma cells and their results suggested that TFA can either stimulate or inhibit cell growth, causing experimental variability to be observed within cell metabolic assays.^61^ Hence there is a need to conduct multiple assays *e.g.*, Live/Dead® staining, to aid preliminary understanding of cell toxicity. Within a biological context the impact of counterions on efficacy, in this case antiviral inhibition, will also be an important consideration for future research. A recent paper by Ardino and colleagues, demonstrated no significant difference in the antibacterial efficacy of TFA and HCl salts of an antibacterial alkylguanidino urea compound.^62^ However in the context of HIV prevention and/or treatment, it must be established if there is any specific interaction between the antiretroviral *e.g.*, cabotegravir and counterion (TFA, HCl) that may limit the drug’s effectiveness. Large-scale *in vivo* studies using simian immunodeficiency virus (SIV) in macaques are the most reliable method currently to establish such a relationship prior to human clinical trials.^63^

An assessment of cell toxicity using concentrations relevant to gel formation *e.g.*, 2% w/v would also be beneficial. Current cell culture methods to estimate cell viability *e.g.*, MTT, MTS alamarBlue®, involve data being quantified by UV or fluorescence plate readers. These methods work more effectively with solubilised peptides rather than hydrogel or precipitated forms of peptides due to interference of peptide precipitates with the UV/fluorescence absorbance/emission signal and difficulty in obtaining a reliable and reproducible background/control with insoluble peptide. Our current setup, using soluble forms of peptides, is representative of concentrations that mimic degradation and dissolution of the peptide hydrogel depot within the body.^11^ As with any new medicinal product, longer-term *in vivo* studies including a full toxicological analysis, immunogenicity,^64^ genotoxicity and carcinogenicity will be required. A recent study by Dekant concluded that the potential for acute TFA toxicity is very low, but that repeated oral exposure to TFA in rats was associated with liver toxicity with mild hypertrophy observed.^65^ The need for longer-term toxicity studies is especially relevant to any long-acting drug delivery system, given it will likely be used chronically in at-risk patients for the prevention or treatment of specific diseases.

### 3.5. *In vitro* drug release

**Fig. 5:**
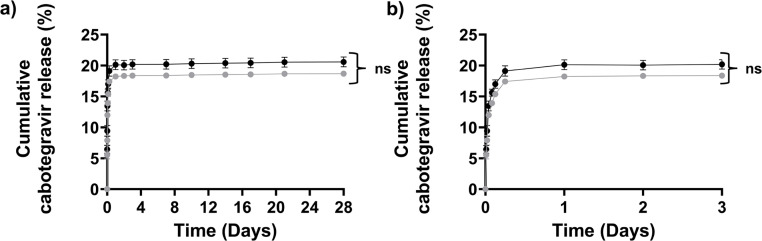
*In vitro* drug release. Cumulative percentage (%) drug release ± SDs of cabotegravir (CAB) from chemically conjugated Napffk(CAB)y(p)G-OH·TFA and Napffk(CAB)y(p)G-OH·HCl hydrogels over (a) 28 days and (b) centered on first 3 days of release (*n* = 4). Key: black circles: cabotegravir release from Napffk(CAB)y(p)G-OH·TFA, grey circles: cabotegravir release from Napffk(CAB)y(p)G-OH·TFA. ns: no significant difference between cabotegravir drug release from drug chemically conjugated to the NapffkyG-OH peptide hydrogels in TFA and HCl salt forms.

A study of drug release *in vitro* provides an estimation of potential release kinetics *in vivo*. Bioequivalence using *in vitro* methods is not always ideal, therefore *in vivo* (animal) and human clinical trials are deemed necessary by regulators in order to link pharmacokinetics and biodistribution to clinical factors such as safety and efficacy. For Napffk(CAB)y(p)G-OH, cabotegravir is covalently conjugated to the hydrogel-forming peptide *via* a labile peptide linker that should cleave under physiological conditions (pH 7.4, H_2_O, 37 °C, esterases). Drug release data collected *in vitro* over 28 days demonstrated no significant difference in the release properties of cabotegravir when comparing HCl and TFA salts of Napffk(CAB)yG-OH (Fig. 5). The majority of drug release is within the first hour of the study (∼13% for both) rising to 18.2% (HCl salt), and 20.1% (TFA salt) within the first 24 hours. This also suggests that a pattern of “burst release” still occurs even after covalent attachment of drug to the peptide. The degree of “burst release” is lower than we previously observed for supramolecular peptide gel systems where the drug is physically encapsulated (∼≥79% for zidovudine + NapffkYG-OH system after 72 hours) and a covalent peptide–drug linker is not present.^15,16^ A lag in cabotegravir release occurs for the remaining 28 days. *In vivo* the peptide–drug linker is likely to be hydrolysed more readily, especially within the presence of a wider range of esterase enzymes. This may result in increased release of drug over long periods of time, however the potential for increased “burst release” must also be considered in any *in vivo* work. Within our *in vitro* studies Napffk(CAB)yG-OH gels are pre-formed 24 hours prior to drug release testing. This does not represent the likely scenario for clinical administration, whereby the formulation would be administered as a solution subcutaneously and a period of gel formation would occur over time. Within future *in vivo* studies, there will be a need to monitor the kinetics of this gelation process and factors such as gel depot shape *e.g.*, *via* ultrasound, that may impact drug release. Once again, rapid gelation is preferred in order to reduce drug “burst release” by diffusion through the hydrogel but also to limit the initial exposure of water to the peptide–drug ester linkage.

Interpretation of the drug release kinetics of cabotegravir and their respective *r*^2^ values showed that both Napffk(CAB)yG-OH·TFA and Napffk(CAB)yG-OH·HCl followed either the Weibull or Korsmeyer–Peppas models (Table S3†). The Korsmeyer–Peppas model has been more widely utilised to define drug release for hydrogel systems and it is likely to be of greater relevance here given its value to our previous work involving peptide hydrogel systems.^15,66^ For both TFA and HCl salts the diffusion exponent *n*, applied within the Korsmeyer–Peppas model, was slightly less than 1 but greater than 0.85 (Table S4†). This corresponds to super case II transport and infers that cabotegravir release from the peptide hydrogel is linked to both cleavage of the peptide–drug ester bond and erosion of the hydrogel matrix.^67^ When utilising the Weibull model (Table S5†) the shape factor *β* defines the mechanism of cabotegravir release. Values of 0.947 (Napffk(CAB)yG-OH·TFA) and 0.950 (Napffk(CAB)yG-OH·HCl), both slightly below 1, indicate cabotegravir release by both diffusion (Fickian diffusion) and hydrogel erosion (case II transport).^68^

The chemical versatility of low molecular weight peptides makes them appealing as advanced drug delivery systems. Their primary sequence can be altered (*e.g.*, functional groups, amino acids, peptide–drug linker) more readily than high molecular weight polymeric systems in order to tailor properties important to their specific application *e.g.*, mechanical properties, sustained release.^48^ Changing the nature of the peptide–drug linker would be particularly interesting given the wide variety of options provided by advances within the area of peptide–, protein– and antibody–drug conjugates, for example amide/ether or carbamate bonds.^69–71^

Whether peptide salt choice has an impact on a formulation drug release properties is likely to be only satisfactorily addressed by comparing pharmacokinetics *in vivo* through bioequivalence studies and as part of optimising the formulations’ future manufacturing upscale. Based on preliminary *in vitro* drug release data alone this does not appear to have a significant impact. However taking into account wider biological considerations may prove significant, if for example a prolonged immune foreign body response to a peptide depot occurred after administration due to or enhanced by the presence of a specific counterion. This may negatively impact drug release if this was to lead to the formation of a dense capsular-like area of fibrous tissue surrounding the depot.^72^

## 4. Conclusions

Our preliminary results demonstrate that the form of salt had no significant impact on properties important to the use of Napffk(CAB)y(p)G-OH supramolecular peptide hydrogels as long-acting injectable drug delivery systems. These characteristics include proteolytic stability, cell cytotoxicity, rheological properties and *in vitro* drug release. This is only a preliminary assessment based on TFA and HCl salts of Napffk(CAB)y(p)G-OH. Given that acetate salts are also widely employed within marketed peptide therapies, it would be appropriate to study acetate salts also within any future work. Additional consideration should be given to the environmental, regulatory and cost implications of counterion choice and how compatible these factors are with existing synthetic processes and manufacturing upscale to cGMP. This is important given that demand for any peptide therapy will increase in size as the clinical trial process proceeds (phase 1–3) and if the peptide is successful licensed as a marketed medicinal product. Outside of those points covered within our discussion, this work raises several relevant questions including how much emphasis should be placed on a pharmaceutical company to provide safety, quality and efficacy data for specific counterions and salt types of new chemical entities? Also what is the future economic and regulatory landscape relating to peptide salts as pharmaceuticals? The majority of current peptides therapies are new drugs under patent and generating revenue to support prior financial research costs. When these patents expire and generic companies look to mimic their formulation will there be any wider intellectual property issues when peptide therapies become off-patent? Could a patent extension be granted with a new salt that is able to demonstrate improved properties? Will a generic company use TFA salts to reduce any associated manufacturing costs? The key next steps for developing our Napffky(p)G-OH platform as a long-acting injectable include: pre-formulation studies, for example salt screening; solubility studies; solution stability; polymorph screening and excipient screening. It will be important to define a maximal concentration for the formulation, below the saturation solubility (mg mL^−1^) of the d-peptide + drug(s) in order to achieve high drug loading and clinically relevant concentrations for ≥84 days.

## Author contributions

J. V. M., E. R. C., Y. A., S. P., S. M. C. and H. S. were involved in data curation, investigation and methodology. J. V. M., S. P. and S. M. C. also contributed to writing – review & editing. G. L. was involved in conceptualisation, data curation, formal analysis, funding acquisition, investigation, methodology, project administration, supervision, writing – original draft and writing – review & editing.

## Conflicts of interest

There are no conflicts to declare.

## Supplementary Material

FD-260-D4FD00194J-s001

## Data Availability

Data for this article, including: proteinase K biostability, oscillatory rheology Napffk(CAB)y(p)G-OH TFA and HCl, MTS cell cytotoxicity Napffk(CAB)y(p)G-OH TFA and HCl; and *in vitro* drug release 28 days CAB release Napffk(CAB)y(p)G-OH TFA and HCl are available [Dataset for “Impact of counterion and salt form on the properties of long-acting injectable peptide hydrogels for drug delivery”] at https://doi.org/10.1039/d4fd00194j.
