# Supplementary material for: Impact of counterion and salt form on the properties of long-acting injectable peptide hydrogels for drug delivery
Source: Faraday Discuss. 2025 Jan 3;260:215–34. doi: 10.1039/d4fd00194j (PMC12076187; doi:10.1039/d4fd00194j)
Supplement: FD-260-D4FD00194J-s001 [file FD-260-D4FD00194J-s001.pdf]

Supplementary Information (SI) for Faraday Discussions.
This journal is © The Royal Society of Chemistry 2024

Impact of counterion and salt form on the properties of long-acting injectable peptide hydrogels for drug delivery

Jessica V. Moore,[a] Emily R. Cross,[a] Yuming An,[a] Sreekanth Pentlavalli,[a] Sophie M. Coulter,[a] Han Sun[a] and Garry Laverty[a*]

[a] Biofunctional Nanomaterials Group, School of Pharmacy, Queen's University Belfast, Medical Biology Centre, 97 Lisburn Road, Belfast, N. Ireland, BT9 7BL.

* Correspondence to garry.laverty@qub.ac.uk

S.1. Synthesis and identification

S.1.1. HPLC purity

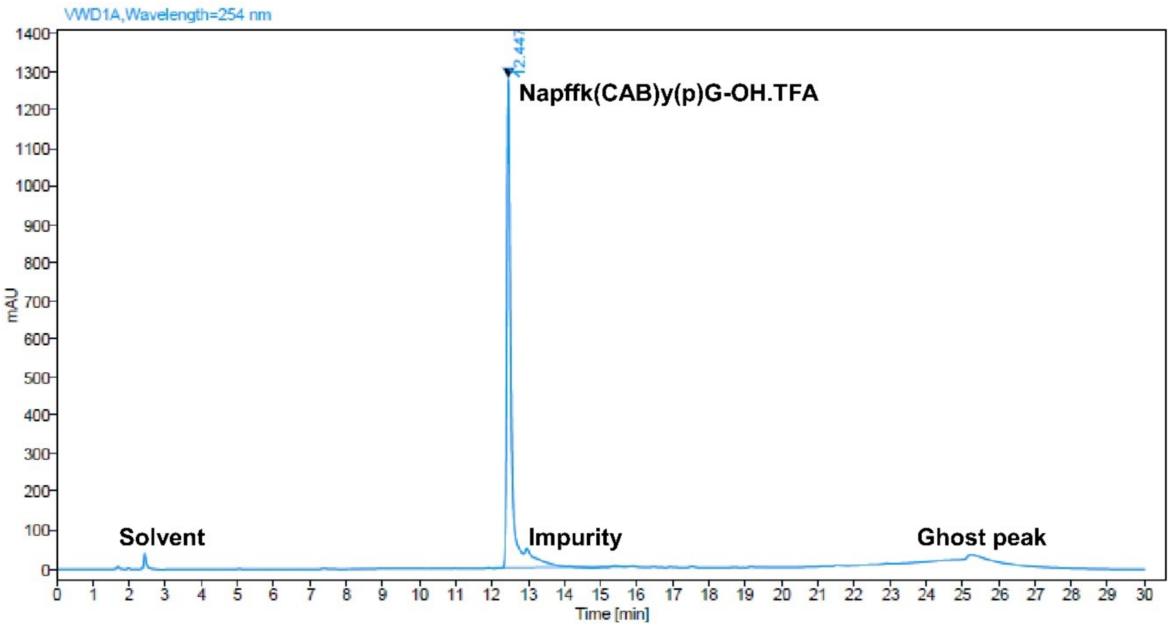

Figure S1. HPLC chromatogram for Napffk(CAB)y(p)G-OH.TFA

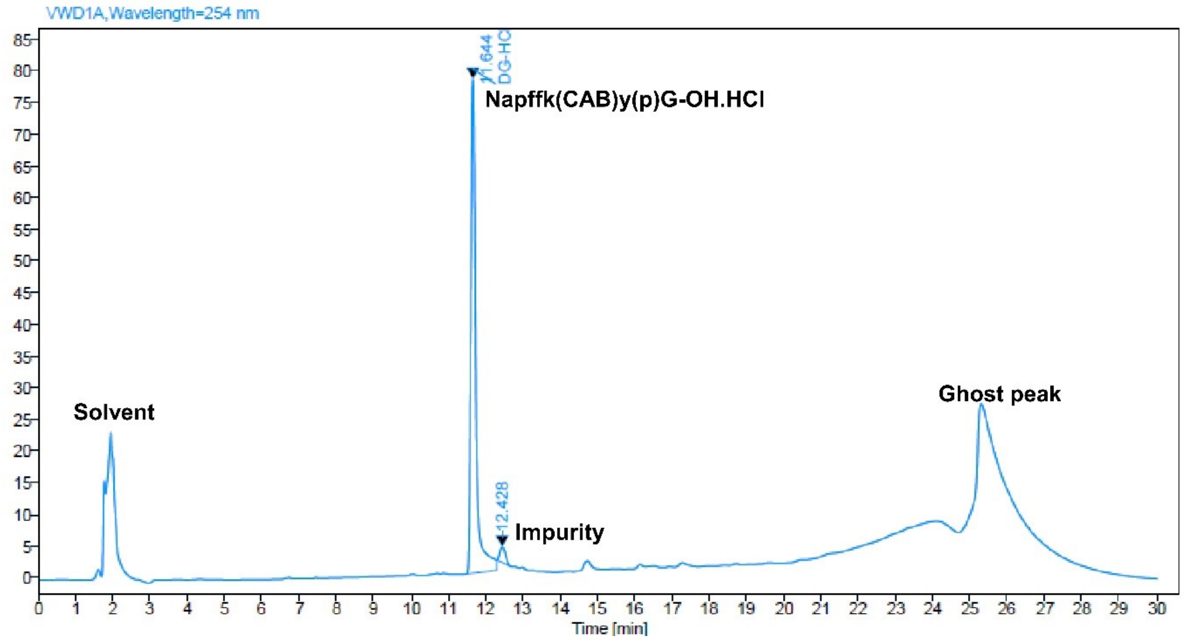

Figure S2. HPLC chromatogram for Napffk(CAB)y(p)G-OH.HCl

S.1.2. Mass spectra

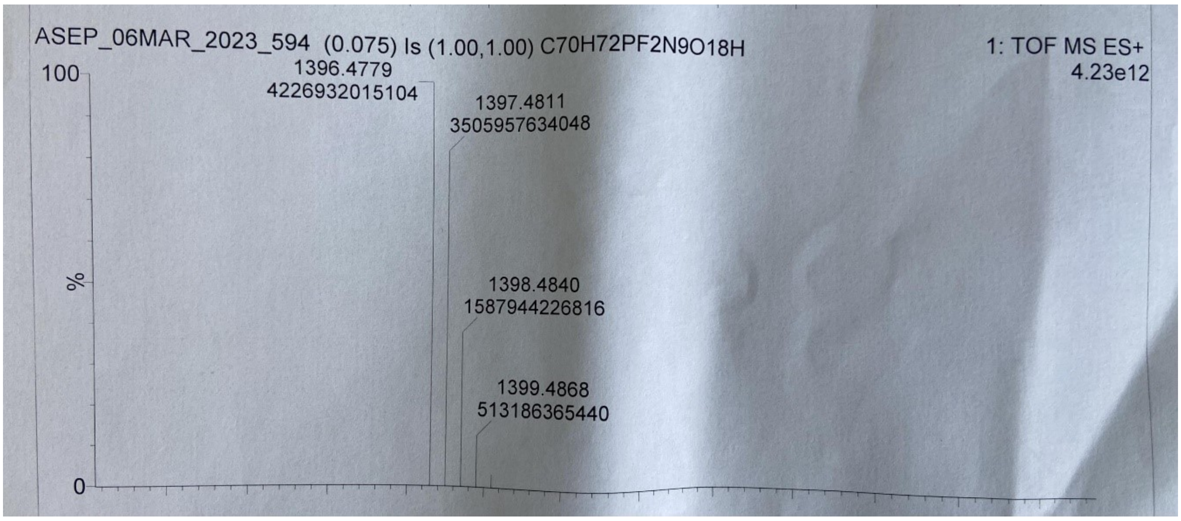

Figure S3. ESI-MS traces for Napffk(CAB)y(p)G-OH.HCl formulated from Napffk(CAB)y(p)G-OH.TFA. Identity confirmed via peaks at 1396 (M) and 1397 (M + H+).

1.3. NMRs

1.3.1. ^{1}H NMRs

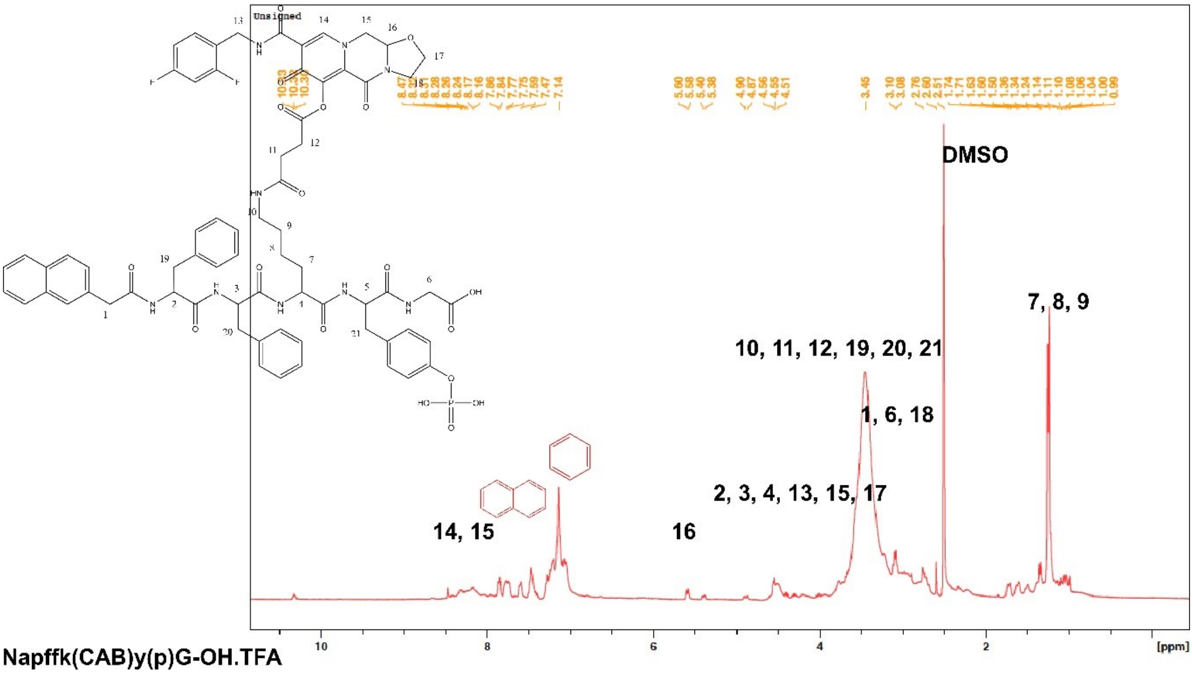

Figure S4. ^{1}H NMR trace for Napffk(CAB)y(p)G-OH.TFA in DMSO-d_6.

^{1}H NMR (C_2D_6OS, TMS standard, 400 MHZ): δ 1.27 (2H, tt, J = 7.4, 7.3 Hz), 1.58 (2H, tt, J = 7.3,

7.0 Hz), 1.94 (2H, q, *J* = 7.4 Hz), 2.67-3.01 (10H, 2.73 (t, *J* = 7.5 Hz), 2.83 (d, *J* = 6.9 Hz), 2.84 (t, *J* = 7.5 Hz), 2.95 (d, *J* = 6.8 Hz), 2.95 (d, *J* = 6.9 Hz)), 3.17 (2H, t, *J* = 7.0 Hz), 3.75 (2H, s), 3.81-4.04 (4H, 3.86 (s), 3.96 (ddd, *J* = 14.5, 7.3, 4.5 Hz)), 4.16-4.42 (5H, 4.24 (dd, *J* = 14.0, 7.0 Hz), 4.26 (ddd, *J* = 15.1, 7.3, 4.5 Hz), 4.37 (t, *J* = 7.5 Hz)), 4.46-4.60 (3H, 4.51 (s), 4.54 (t, *J* = 6.9 Hz)), 4.60-4.72 (2H, 4.66 (t, *J* = 6.9 Hz), 4.66 (t, *J* = 6.8 Hz)), 5.29 (1H, dd, *J* = 9.9, 4.0 Hz), 6.83 (1H, dd, *J* = 8.4, 1.6 Hz), 6.91-7.08 (4H, 6.97 (ddd, *J* = 8.3, 1.6, 0.5 Hz), 7.02 (ddd, *J* = 8.3, 1.2, 0.5 Hz)), 7.14-7.35 (10H, 7.20 (tt, *J* = 7.7, 1.5 Hz), 7.20 (tt, *J* = 7.7, 1.5 Hz), 7.26 (dddd, *J* = 7.8, 1.5, 1.2, 0.5 Hz), 7.26 (dddd, *J* = 7.8, 1.5, 1.2, 0.5 Hz), 7.28 (tdd, *J* = 7.7, 1.9, 0.5 Hz), 7.28 (tdd, *J* = 7.7, 1.9, 0.5 Hz)), 7.36-7.66 (5H, 7.41 (dd, *J* = 1.6, 0.5 Hz), 7.44 (dddd, *J* = 8.0, 6.9, 1.8, 0.5 Hz), 7.46 (dd, *J* = 8.4, 0.5 Hz), 7.56 (dddd, *J* = 7.9, 6.9, 1.7, 0.5 Hz), 7.60 (ddd, *J* = 8.4, 2.0, 0.4 Hz)), 7.68-8.00 (4H, 7.75 (ddddt, *J* = 7.9, 1.8, 1.5, 0.5, 0.4 Hz), 7.80 (tq, *J* = 1.9, 0.5 Hz), 7.89 (dddt, *J* = 8.0, 1.9, 1.7, 0.5 Hz), 7.93 (ddq, *J* = 8.4, 1.5, 0.5 Hz)), 8.51 (1H, s).

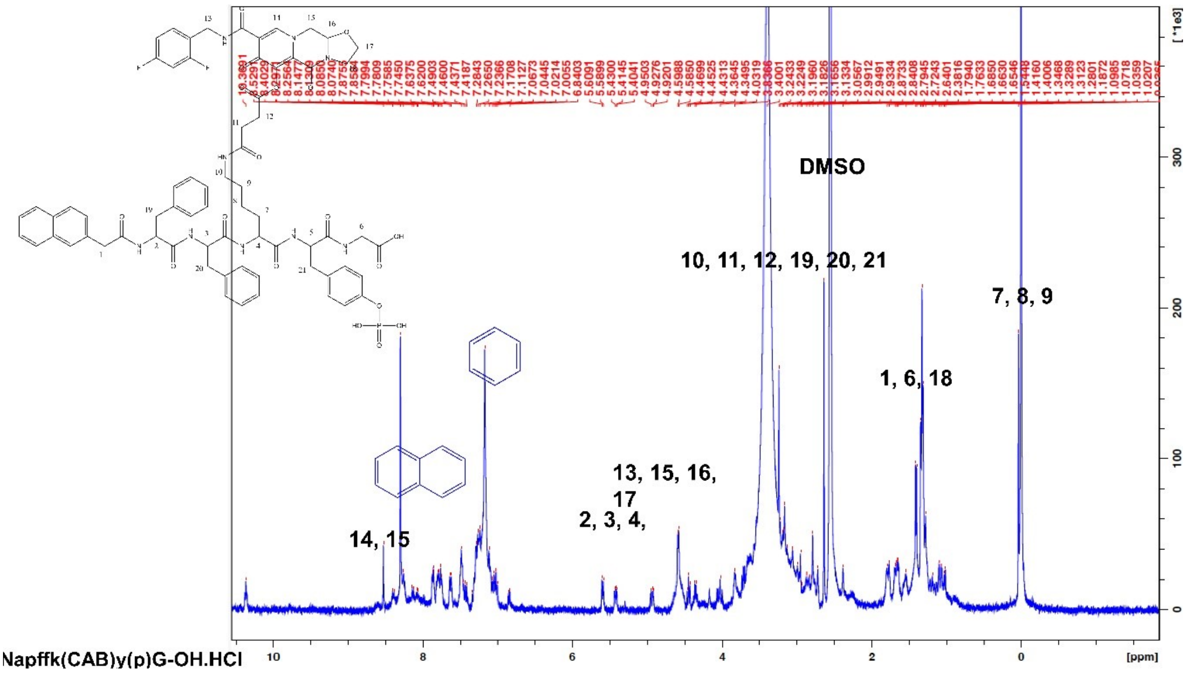

Figure S5. ^{1}H NMR trace for Napffk(CAB)y(p)G-OH.HCl in DMSO-d_6.

^{1}H NMR (C_2D_6OS, TMS standard, 400 MHZ): δ 1.27 (2H, tt, *J* = 7.4, 7.3 Hz), 1.58 (2H, tt, *J* = 7.3, 7.0 Hz), 1.94 (2H, q, *J* = 7.4 Hz), 2.67-3.01 (10H, 2.73 (t, *J* = 7.5 Hz), 2.83 (d, *J* = 6.9 Hz), 2.84 (t, *J* = 7.5 Hz), 2.95 (d, *J* = 6.8 Hz), 2.95 (d, *J* = 6.9 Hz)), 3.17 (2H, t, *J* = 7.0 Hz), 3.75 (2H, s), 3.81-4.04 (4H, 3.86 (s), 3.96 (ddd, *J* = 14.5, 7.3, 4.5 Hz)), 4.16-4.42 (5H, 4.24 (dd, *J* = 14.0, 7.0 Hz), 4.26 (ddd, *J* = 15.1, 7.3, 4.5 Hz), 4.37 (t, *J* = 7.5 Hz)), 4.46-4.60 (3H, 4.51 (s), 4.54 (t, *J* = 6.9 Hz)), 4.60-4.72 (2H, 4.66 (t, *J* = 6.9 Hz), 4.66 (t, *J* = 6.8 Hz)), 5.29 (1H, dd, *J* = 9.9, 4.0 Hz), 6.83 (1H, dd, *J* = 8.4, 1.6 Hz), 6.91-7.08 (4H, 6.97 (ddd, *J* = 8.3, 1.6, 0.5 Hz), 7.02 (ddd, *J* = 8.3, 1.2, 0.5 Hz)), 7.14-7.35 (10H, 7.20 (tt, *J* = 7.7, 1.5 Hz), 7.20 (tt, *J* = 7.7, 1.5 Hz), 7.26 (dddd, *J* = 7.8, 1.5, 1.2, 0.5 Hz),

7.26 (dddd, *J* = 7.8, 1.5, 1.2, 0.5 Hz), 7.28 (tdd, *J* = 7.7, 1.9, 0.5 Hz), 7.28 (tdd, *J* = 7.7, 1.9, 0.5 Hz)), 7.36-7.66 (5H, 7.41 (dd, *J* = 1.6, 0.5 Hz), 7.44 (dddd, *J* = 8.0, 6.9, 1.8, 0.5 Hz), 7.46 (dd, *J* = 8.4, 0.5 Hz), 7.56 (dddd, *J* = 7.9, 6.9, 1.7, 0.5 Hz), 7.60 (ddd, *J* = 8.4, 2.0, 0.4 Hz)), 7.68-8.00 (4H, 7.75 (ddddt, *J* = 7.9, 1.8, 1.5, 0.5, 0.4 Hz), 7.80 (tq, *J* = 1.9, 0.5 Hz), 7.89 (dddt, *J* = 8.0, 1.9, 1.7, 0.5 Hz), 7.93 (ddq, *J* = 8.4, 1.5, 0.5 Hz)), 8.51 (1H, s).

1.3.2. ^{31}P NMRs

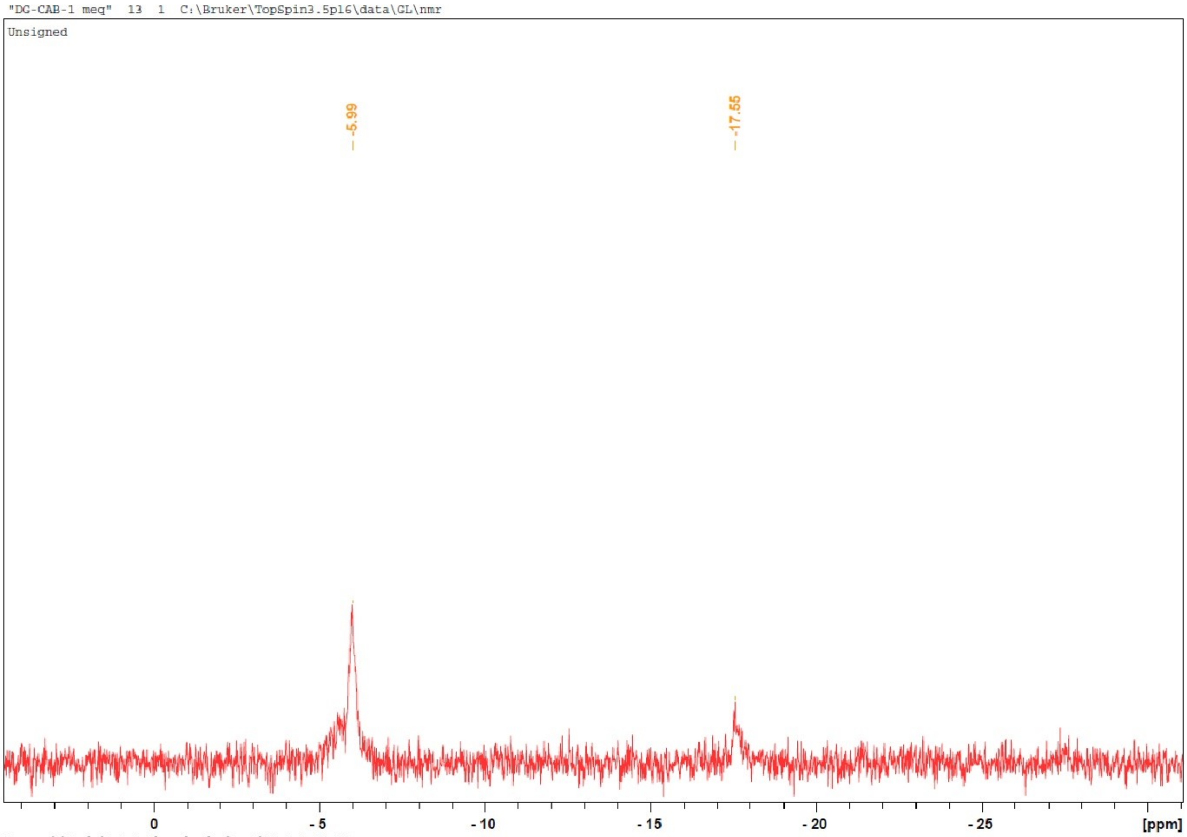

Napffk(CAB)y(p)G-OH.TFA

Figure S6. ^{31}P NMR trace for Napffk(CAB)y(p)G-OH.TFA, peak at 5.99 demonstrates the presence and retention of the phosphate grouping on the tyrosine motif.

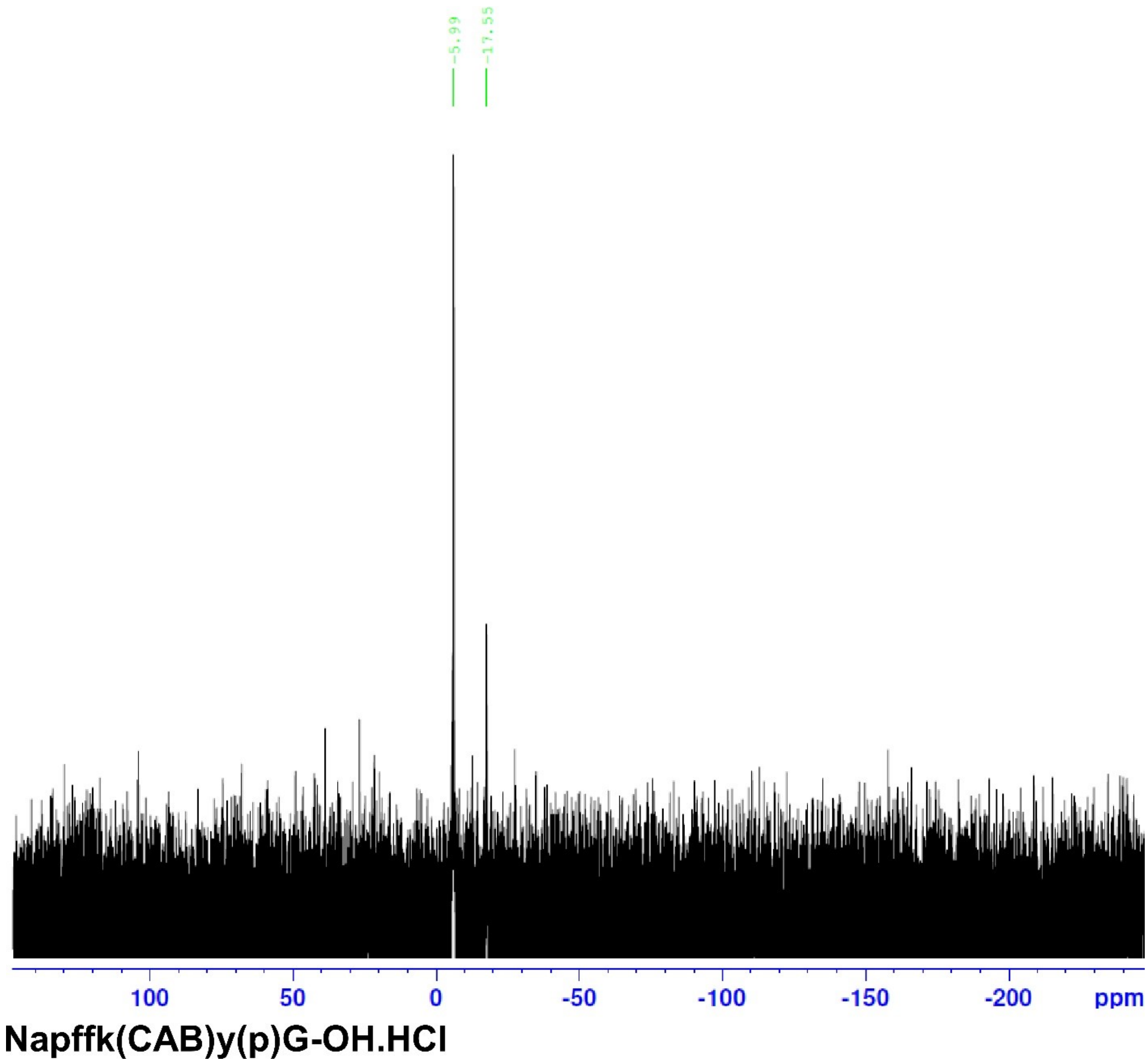

Figure S7. ^{31}P NMR trace for Napffk(CAB)y(p)G-OH.HCl, peak at 5.99 demonstrates the presence and retention of the phosphate grouping on the tyrosine motif.

S.2. Mechanical properties

Table S1. Stepwise formulation of a self-assembling enzyme-triggered gelator using 2% w/v Napffk(CAB)y(p)G-OH as an example (final volume 500 µL).

Formulation Step	Constituent	Quantity added
1	Napffk(CAB)y(p)G-OH	10 mg pre-weighed in HPLC vial
2	1.0 M NaOH	10 µL
3	PBS	200 µL[a)]
4	1.0 M NaOH	10 µL (according to pH, keep to 7.4)
5	PBS	200 µL[a)]
6	PBS	to final volume (500 µL)[a)]
7	Alkaline phosphatase	2 U (2 µL)[b)]

[a)] Sonicate (30 minutes) using a Branson 3510 sonic bath (Branson Ultrasonics Danbury, Connecticut, USA). Then the pH was monitored using a pH probe.

[b)] Overnight incubation at 37°C.

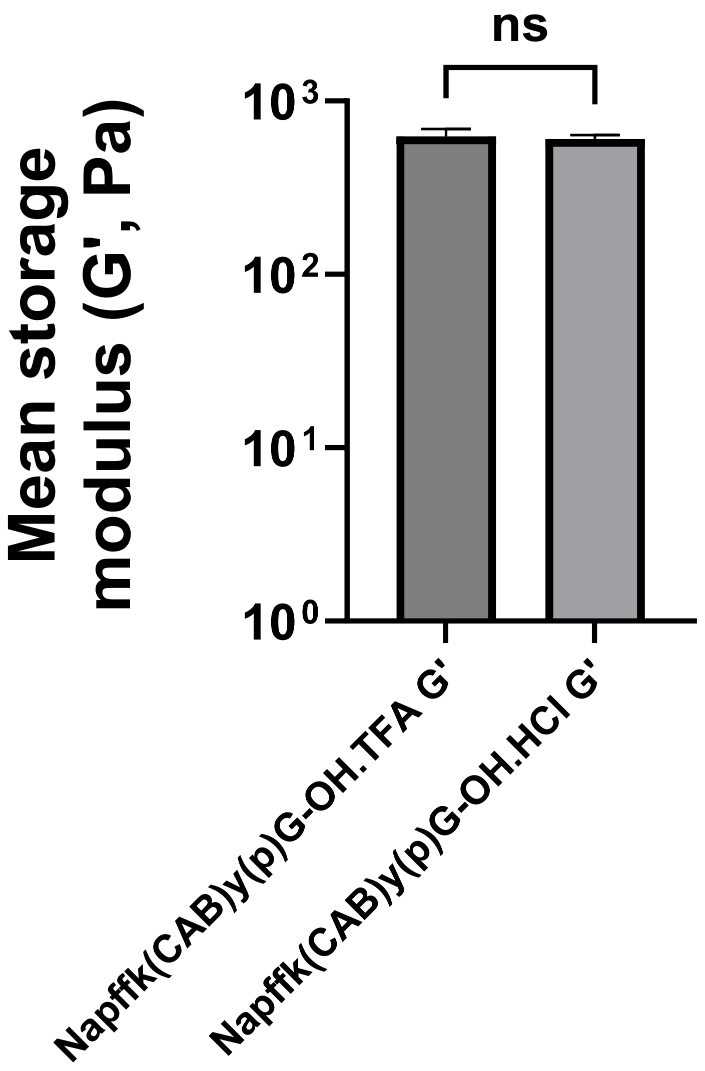

Figure S8. Mean value of storage modulus (G′) for each peptide hydrogel salt at 2% w/v derived from Figure 3a, individual frequency sweeps (1 – 100 rad/s, strain = 0.5%) providing a comparison of gel stiffness. ****p < 0.0001 difference in G′.

Table S2. Gelation times for peptide hydrogels upon addition of 3.98 U/mL alkaline phosphatase enzyme derived from data in Figure 3 c – e.

**Peptide**	**G′ and G″ cross**	**G′ > 2x G″**	**Time for stable G′**
Napffk(CAB)y(p)G-OH.TFA	1.67 mins	12.5 mins	65.3 mins
Napffk(CAB)y(p)G-OH.HCl	1.67 mins	13.8 mins	62.7 mins

S.3. Cell cytotoxicity

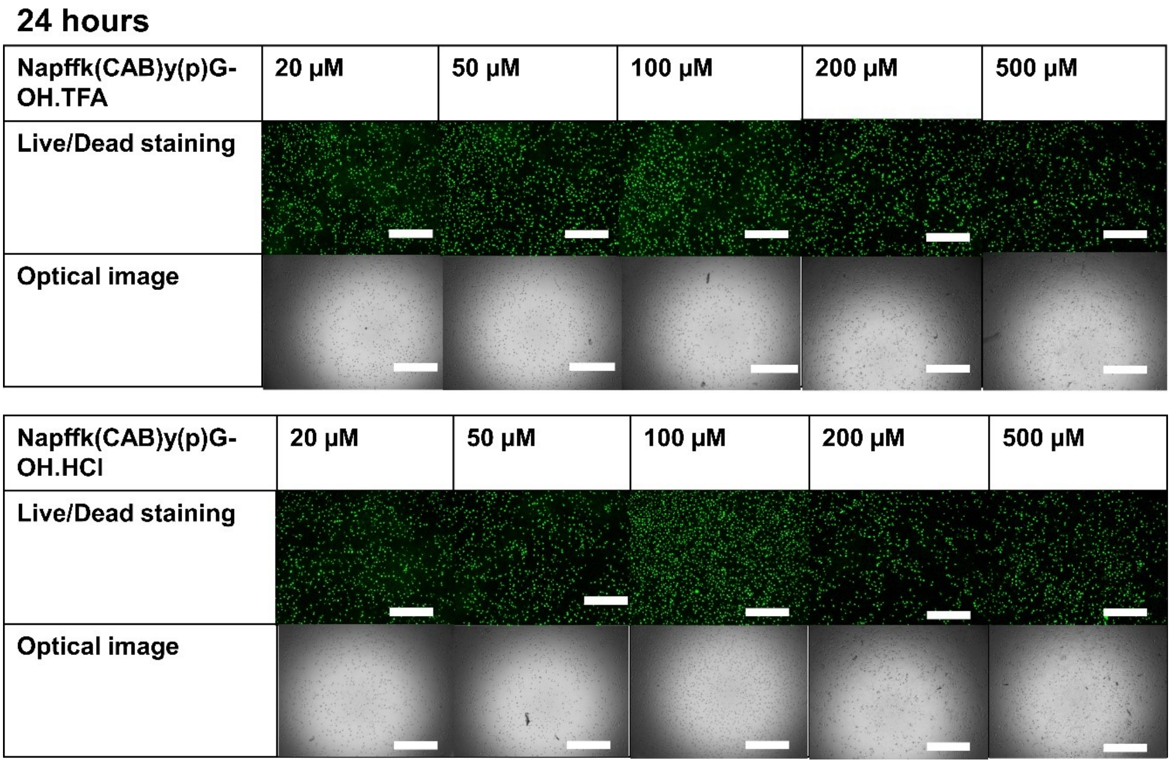

Figure S9. Live/Dead® staining of fully solubilized Napffk(CAB)y(p)G-OH.TFA and Napffk(CAB)y(p)G-OH.HCl at a concentration range of 20 – 500 μM (24 hours, scale bar: 300 μm).

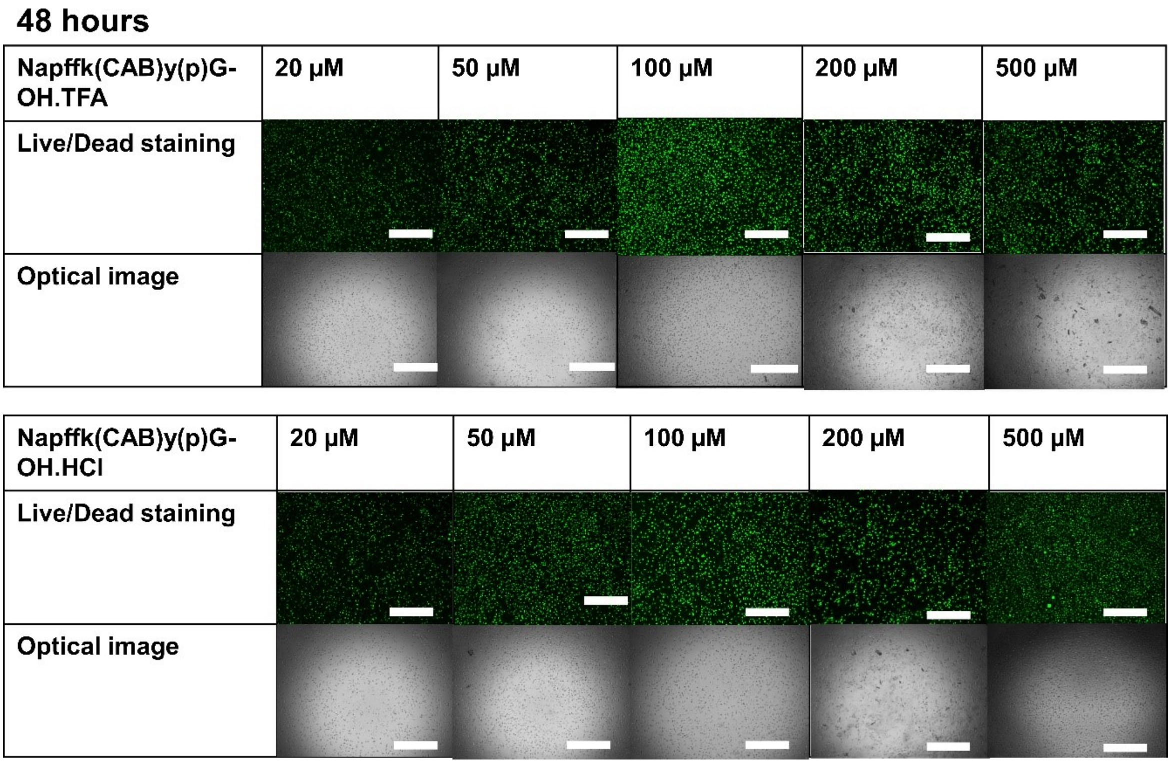

Figure S10. Live/Dead® staining of fully solubilized Napffk(CAB)y(p)G-OH.TFA and Napffk(CAB)y(p)G-OH.HCl at a concentration range of 20 – 500 µM (48 hours, scale bar: 300 µm).

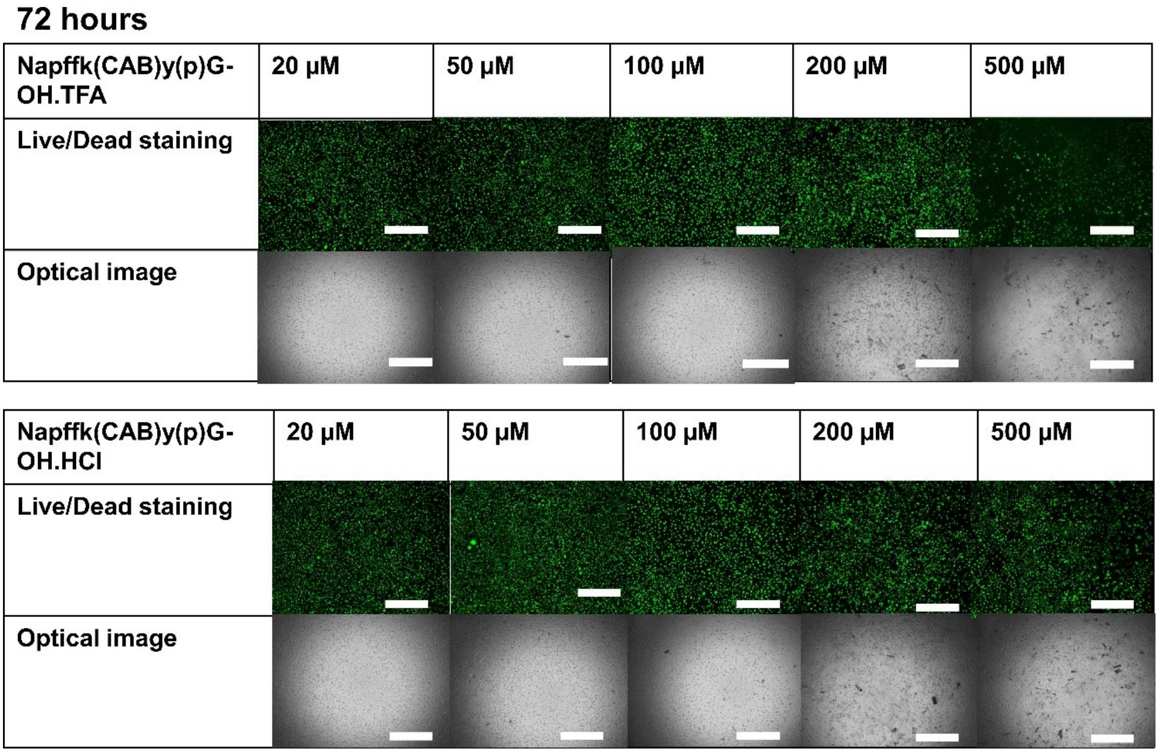

Figure S11. Live/Dead® staining of fully solubilized Napffk(CAB)y(p)G-OH.TFA and Napffk(CAB)y(p)G-OH.HCl at a concentration range of 20 – 500 µM (72 hours, scale bar: 300 µm).

S.4. *In vitro* drug release

Preparation of cabotegravir standards: A cabotegravir stock solution (1 mg/mL) was prepared in Milli-Q water and diluted to the final concentrations required for the standard calibration curve (9 concentrations across 0.195 – 50 µg/mL). The concentration of cabotegravir standards were determined using an Agilent 1260 Series analytical HPLC system (Agilent Technologies Ltd, Cork, Ireland) fitted with a Gemini C_{18} column (250 x 4.6 mm, 5 µm particle size, 110 Å; Phenomenex, Macclesfield, UK) and UV detector (Wavelength: 256 nm). A mobile phase consisting of acetonitrile (ACN) and water (ACN: H_2O 60: 40) was employed at a flow of 1 mL/min. The retention time of cabotegravir was 2.2 mins using this setup.

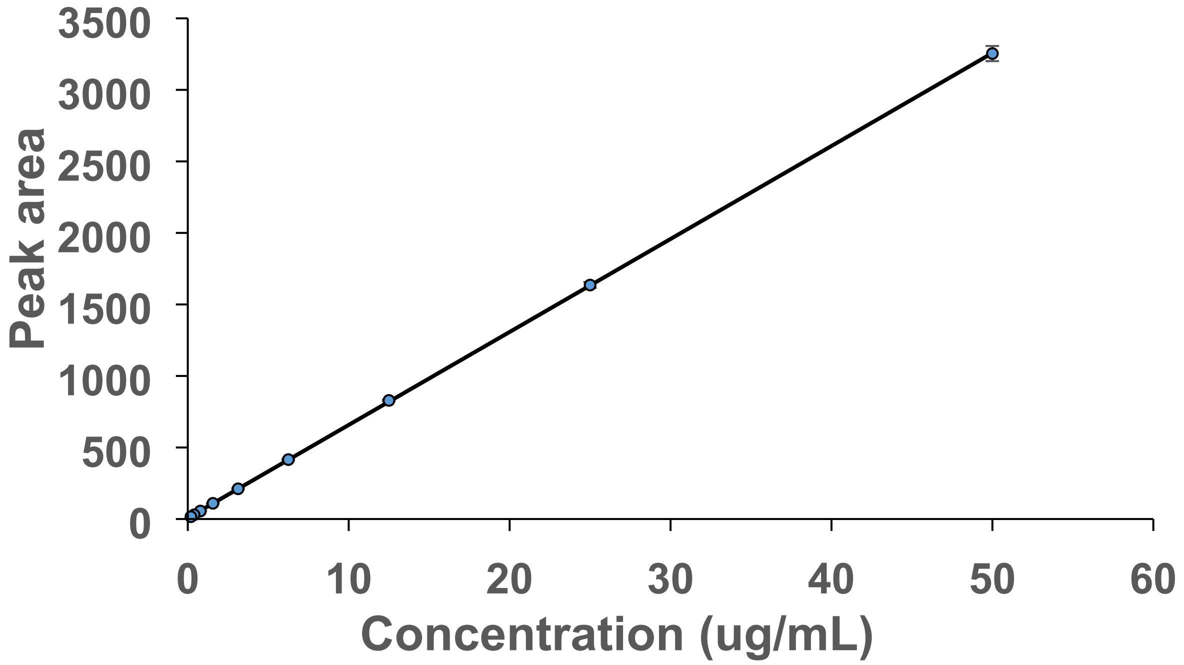

Figure S12. Calibration curve developed for cabotegravir between 0.195 – 50 µg/mL (*n* = 3).

Table S3. Model fitting performed using KinetDS 3.0 rev. 2010 software with the r^2 value displayed for each model.

**Formulation**	**Model**					
	**Zero order**	**First order**	**Kormeyers-Peppas**	**Weibull**	**Hixson-Crowell**	**Higuchi**
	r^2	r^2	r^2	r^2	r^2	r^2
Napffk(CAB)yG-OH.TFA	0.2439	0.0471	0.8584	0.8604	0.1291	-1.7928
Napffk(CAB)yG-OH.HCl	0.2408	0.0464	0.8565	0.8586	0.1245	-1.9646

Table S4. The parameters fitted with the Kormeyers-Peppas model of drug release using KinetDS software for 28 day release profiles for each formulation.

**Formulation**	r^2	k	n
Napffk(CAB)yG-OH.TFA	0.8584	11.51±0.644	0. 942±0.102
Napffk(CAB)yG-OH.HCl	0.8565	12.78±0.651	0.945±0.103

Table S5. The parameters fitted with the Weibull model of drug release using KinetDS software for 28 day release profiles for each formulation.

**Formulation**	r^2	α	β
Napffk(CAB)yG-OH.TFA	0.8604	7.969±0.642	0.947±0.102
Napffk(CAB)yG-OH.HCl	0.8586	7.105±0.649	0.950±0.103
